# Effects of Dietary Baicalin on Growth Performance, Serum Biochemical Parameters, Liver Health, Intestinal Health, and Microbiota of Yellow Catfish (*Pelteobagrus fulvidraco*)

**DOI:** 10.3390/ani15192903

**Published:** 2025-10-04

**Authors:** Haonan Liu, Xinru Li, Yang Fan, Yang Xiao, Yunfeng Chen, Xiaoqin Li, Xiangjun Leng

**Affiliations:** 1 National Demonstration Center for Experimental Fisheries Science Education, Shanghai Ocean University, Shanghai 201306, China; haonanliu2002@163.com (H.L.); m15237303262@163.com (X.L.); 19850226601@163.com (Y.F.); xy2000224@163.com (Y.X.); chenyf256893@163.com (Y.C.); 2 Research Centre of the Ministry of Agriculture and Rural Affairs on Environmental Ecology and Fish Nutrition, Shanghai Ocean University, Shanghai 201306, China

**Keywords:** baicalin, *Pelteobagrus fulvidraco*, growth performance, antioxidant levels, liver and intestinal health, intestinal microbiota

## Abstract

This study investigated the effects of dietary baicalin on the growth and health of yellow catfish (*Pelteobagrus fulvidraco*). Five diets with the addition of 0 (control), 100, 200, 400, and 800 mg/kg of baicalin were fed to juvenile yellow catfish (11.19 ± 0.07 g) for 56 days. Dietary supplementation with 200 or 400 mg/kg baicalin increased weight gain (*p* < 0.05) and reduced the feed conversion ratio (FCR) (*p* < 0.05). The BA400 group exhibited higher total antioxidant capacity, catalase, superoxide dismutase activity, and lower malondialdehyde level in the liver and intestine than the control (*p* < 0.05). In the 400 mg/kg baicalin group, the hepatic vacuolization rate was lower (*p* < 0.05), and the intestinal villus width and villus height were higher than those in the control (*p* < 0.05). In terms of intestinal microbiota composition, the 400 mg/kg baicalin group demonstrated lower abundances of *Proteobacteria* and *Actinobacteria* (phylum level) and *Cetobacterium*, *Candidatus_Arthromitus*, and *Plesiomonas* (genus level) than the control (*p* < 0.05), while the relative abundances of *Fusobacteriota* and *Lactobacillus* (ZOR0006) increased in this group (*p* < 0.05). In conclusion, dietary baicalin supplementation significantly improved the growth, antioxidant capacity, and intestinal morphology and positively modulated the intestinal microbiota composition of yellow catfish. The optimal dietary supplementation level of baicalin is 400 mg/kg for yellow catfish.

## Simple Summary

Baicalin is the major bioactive compound in the Chinese herb *Scutellaria baicalensis*, and its positive effects have been reported in some fish. The present study investigated the effects of dietary baicalin on the growth and health of yellow catfish (*Pelteobagrus fulvidraco*). The results indicated that dietary baicalin supplementation significantly improved the growth performance, antioxidant capacity, and morphology of the liver and intestine, as well as positively modulated the intestinal microbiota composition of yellow catfish. The optimal inclusion of dietary baicalin was 400 mg/kg. This finding will guide the application of baicalin in aquatic feeds.

## 1. Introduction

Baicalin (C_21_H_18_O_11_), a yellow bitter flavonoid extracted from the roots of *Scutellaria baicalensis*, is a key bioactive compound in this Chinese medicinal herb [1]. It exhibits various pharmacological effects, including antioxidant, anti-inflammatory, immune-enhancing, antibacterial, hepatoprotective, and intestinal protective properties [2]. Baicalin has been shown to improve the growth performance, antioxidant capacity, and immune function in land animals such as preweaned calves [3], broilers [4], ducks [5], and piglets [6]. In aquatic animals, baicalin was reported to enhance the growth performance, antioxidant capacity, and resistance to hepatic oxidative stress and *Streptococcus agalactiae* infection in Nile tilapia (*Oreochromis niloticus*) [7,8]. Baicalin has also been shown to protect giant river prawns (*Macrobrachium rosenbergii*) against *Vibrio parahaemolyticus* [9] and strengthen the ability of yellow catfish (*Pelteobagrus fulvidraco*) to resist *Aeromonas hydrophila* infection [10]. Additionally, baicalin yeast culture improved the growth performance, antioxidant capacity, and immune function in giant grouper (*Epinephelus lanceolatus*) [11].

The yellow catfish is an important freshwater aquaculture species in China, valued for its high market demand and nutritional quality. However, in intensive aquaculture systems, farmed fish are generally susceptible to physiological challenges such as oxidative stress and inflammatory responses due to high stocking density and environmental fluctuations. The carnivorous nature and high dietary lipid requirements in this fish suggest a heightened potential for metabolic disorders such as hepatic steatosis. These concerns highlight the need for safe and effective feed additives to enhance growth and health management. Although previous studies have demonstrated that active compounds in Chinese herbal medicine, such as emodin [12], berberine [13], *Ganoderma lucidum* polysaccharides [14], and *Astragalus* polysaccharides [15], could enhance the growth performance and immune function or modulate intestinal microbiota composition in yellow catfish, their application ranges and functional scope remain limited. There is continued interest in identifying novel additives with broader efficacy and better safety profiles. Therefore, this study aims to explore the effects of dietary supplementation of baicalin on growth performance, morphometric indices, serum biochemical parameters, hepatic and intestinal antioxidant capacity, liver health, and intestinal health and microbiota in yellow catfish. The findings will guide the application of baicalin in aquatic feeds.

## 2. Materials and Methods

All experimental animal care protocols underwent approval by Shanghai Ocean University’s Institutional Animal Care and Use Committee (Approval No. SHOU-DW-2024-140). Experimental operations were rigorously conducted in compliance with the standardized ethical regulations promulgated by Shanghai Ocean University’s Animal Ethics Oversight Committee.

### 2.1. Experimental Design and Diets

Five isonitrogenous (46% crude protein) and isolipidic (8.9% crude lipid) diets were formulated using fish meal, soybean meal, and chicken meal as protein sources. According to the previous studies [7,8,10,16], baicalin (purity: 95%, purchased from Macklin Biochemical Technology Co., Ltd., Shanghai, China) was added at concentrations of 0, 100, 200, 400, and 800 mg/kg (BA0, BA100, BA200, BA400, and BA800), respectively. Accordingly, the wheat flour inclusion was reduced to balance the formulation, while the proportions of the other ingredients remained constant. All feed ingredients were ground to pass through a 60-mesh sieve, then thoroughly mixed and extruded to form slow-sinking pellets (2.0 mm diameter) using a single-screw extruder (LX-75, Longxiang Food Machinery Factory, Xingtai, China) with a pelleting temperature of 90 °C. The pellets were air-dried at 60 °C and stored in a cool, dry environment. The diet formulations and nutritional compositions are shown in Table 1.

### 2.2. Experimental Fish and Feeding Management

A total of 800 yellow catfish (initial average body weight: 4.15 g) were obtained from Suyu Aquaculture Company (Huzhou, China) and then fed with the control diet for 4 weeks in cement pools at the Binhai Aquaculture Base of Shanghai Ocean University (Shanghai, China). After the acclimation, 225 healthy fish (average weight: 11.19 ± 0.07 g) were selected and randomly stocked into 15 cages (1.0 m × 1.0 m × 1.2 m; mesh size: 0.3 mm; material: Nylon; 15 fish per cage) with 5 treatments and 3 replicates per treatment. Three independent pools with the same water source and management were employed, and each pool contained five cages, which were randomly assigned to the five treatments.

The feeding protocol was conducted with reference to the description by Xu et al. [16] and strictly controlled to ensure consistency across all experimental groups. The daily ration was set at 3% to 6% of the total fish body weight per cage and was adjusted weekly based on collective weighing. Every morning, the feed was weighed to ensure the same amount for each treatment group. The slow-sinking feed was offered by hand twice daily (08:00 and 18:00). To minimize waste and ensure precise intake, the allotted feed for each cage was divided into small batches and introduced manually over 10 min, which has been determined through the pre-trial observation. The uneaten feed settling on the bottom was carefully siphoned out within 30 min and then dried and weighed to calculate the actual feed consumption.

Water quality was detected as follows: temperature 25–30 °C, dissolved oxygen > 5 mg/L, pH 7.5–8.0, ammonia nitrogen < 0.2 mg/L, and nitrite < 0.1 mg/L. One-third of the water was replaced twice weekly with filtered pond water. The feeding trial was conducted at the Binhai Aquaculture Base of Shanghai Ocean University for 56 days.

### 2.3. Sample Collection

At the end of the feeding trial, the fish were starved for 24 h, and then total weight and fish number per cage were recorded to calculate survival, weight gain (WG), specific growth rate (SGR), and feed conversion ratio (FCR). Before sampling, fish were anesthetized with MS-222 anesthetic. Three fish per cage were randomly selected and stored at −20 °C for whole-body proximate composition analysis. Another three fish were measured for body length and weight to calculate condition factors (CFs). Subsequently, blood (~1 mL) was collected from the caudal vein of the largest three individuals (45–55 g) from each cage using a 1 mL medical syringe. Serum was centrifuged at 3000 rpm for 10 min; then, approximately 1.3 mL of serum was collected per replicate and stored at −80 °C for biochemical assays. After sampling blood, the fish were dissected, and then the viscera and liver weights were recorded to calculate the viscerosomatic index (VSI) and hepatosomatic index (HSI). Liver and intestinal tissues were stored at −80 °C for antioxidant analysis. Liver and intestinal samples from BA0, BA400, and BA800 groups were fixed in Bouin’s solution for H&E staining and histological evaluation. Three fish per cage were quickly dissected, and the hind intestinal contents from the BA0 and BA400 groups were collected aseptically and placed into enzyme-free freezing tubes for preservation at −80 °C to analyze the microbiota community.

### 2.4. Measurement Indicators and Methods

#### 2.4.1. Growth Performance and Body Morphometric Indices


SR (%) = (final number of fish/initial number of fish) × 100

WG (%) = [(final body weight − initial body weight)/initial body weight] × 100

SGR (%) = 100 × [ln(final body weight) − ln(initial body weight)]/experiment days

FCR=feed intake (g)/weight gain (g)

HSI (%) = [final liver weight (g)/final body weight (g)] × 100

VSI (%) = [final visceral weight (g)/final body weight (g)] × 100

$$CF\;=\;final\;body\;weight\;(g)/body\;length\;{\left(cm\right)}^{3}\;\times \;100$$

#### 2.4.2. Proximate Composition of Diets and Whole Body

Referring to the AOAC method, the moisture content in feed and whole fish was determined by oven drying at 105 °C, and crude protein was analyzed using the Kjeldahl nitrogen analyzer (2300-Auto-Analyzer, Foss Tecator, Höganäs, Sweden), while ash content was quantified by combustion at 550 °C in a muffle furnace (SXL-1008, Shanghai Jinhong Experimental Equipment Co., Shanghai, China). Crude fat was measured by the chloroform–methanol assay [17].

#### 2.4.3. Serum, Hepatic, and Intestinal Biochemical Parameters

The liver and intestinal tissues were homogenized at 10,000 rpm for 10 s in ice-cold physiological saline (1:9 *w*/*v*) and then centrifuged at 3000 rpm for 10 min (4 °C). The supernatants were collected and stored at −80 °C for subsequent analyses.

The measurements of total cholesterol (TCHO; COD-PAP assay), triglycerides (TGs; GPO-PAP assay), total protein (TP; Coomassie brilliant blue assay), lysozyme (LZM; turbidimetric assay), alkaline phosphatase and acid phosphatase (ALP and ACP; microplate enzymatic assay), alanine aminotransferase and aspartate aminotransferase (ALT and AST; microplate colorimetric assay), total antioxidant capacity (T-AOC; FRAP assay), malondialdehyde (MDA; TBA assay), catalase (CAT; ammonium molybdate colorimetry assay), and superoxide dismutase (SOD; hydroxylamine oxidation assay) were performed using commercial kits (Nanjing Jiancheng Bioengineering Institute, Nanjing, China).

#### 2.4.4. Histological Examination

The liver and intestinal tissues from the BA0, BA400, and BA800 groups underwent ethanol dehydration, xylene clearing, and paraffin embedding. Sectioned samples were stained with hematoxylin–eosin (H&E) and mounted for microscopic observation. Histomorphological features were captured using optical microscopy (Olympus DP71), and the vacuolization area quantification and intestinal morphology measurements were performed through ImageJ 1.53 software.

#### 2.4.5. Intestinal Microbiota Analysis Based on 16S rRNA

Intestinal content DNA from the BA0 and BA400 groups was extracted using QIAamp DNA Stool Mini Kit (Qiagen, Venlo, The Netherlands). After purity verification (NanoDrop 2000, Thermo Scientific, Wilmington, DE, USA), the V3–V4 hypervariable regions of bacterial 16S rRNA genes were amplified and sequenced on the Illumina NovaSeq 6000 platform (2×250 bp paired-end reads). Raw sequences were processed through the QIIME2 pipeline with DADA2 denoising, chimera removal, and 100% similarity clustering. Operational taxonomic units (OTUs) were taxonomically classified against the SILVA 138 database. Alpha diversity indices and KEGG functional predictions were generated using PICRUSt2. Sequencing services were provided by Shanghai Shunshi Biotechnology Co., Ltd. (Shanghai, China).

### 2.5. Statistical Analysis

All data were expressed as mean ± standard deviation (SD). One-way ANOVA was performed using SPSS 26.0. Tukey’s HSD test was used for multiple comparisons. A *p*-value below 0.05 was considered statistically significant. Graphical visualizations were created with GraphPad Prism 9.0 (GraphPad Software) and Microsoft Excel 2021.

## 3. Results

### 3.1. Growth Performance and Morphometric Indices

Compared with the control group (BA0), the final weight, survival, WG, and SGR of the BA200 and BA400 groups were significantly higher (*p* < 0.05), along with a significantly lower FCR (*p* < 0.05). No significant differences were observed in growth performance among the BA100, BA800, and control (BA0) groups (*p* > 0.05) except for survival. CF, VSI, and HSI showed no significant differences among all the groups (*p* > 0.05) (Table 2).

### 3.2. Serum Biochemical Indices

In Table 3, serum TG levels in the BA100 and BA400 groups, as well as AST activity across all baicalin-supplemented groups, were significantly lower than those in the control group (BA0) (*p* < 0.05). The BA400 group displayed significantly elevated ALP activity, while both the BA200 and BA400 groups showed higher LZM activity than the control (*p* < 0.05). No significant differences were detected in TCHO, TP content, or ALT activity among all the groups (*p* > 0.05).

### 3.3. Antioxidant Capacity

As shown in Table 4, the T-AOC, CAT, and SOD activities in liver and intestine increased firstly and then decreased with the increasing dietary baicalin levels, peaking in the BA400 group with significantly higher values than the control (*p* < 0.05). The BA400 group also presented significantly lower MDA content than the control (*p* < 0.05). Intestinal LZM activity showed no differences among groups (*p* > 0.05).

### 3.4. Whole-Body Proximate Composition

There were no significant differences in whole-body composition among all groups (*p* > 0.05), including moisture, crude ash, crude lipid, and crude protein contents (Table 5).

### 3.5. Liver and Intestinal Histomorphology

**Figure 1:**
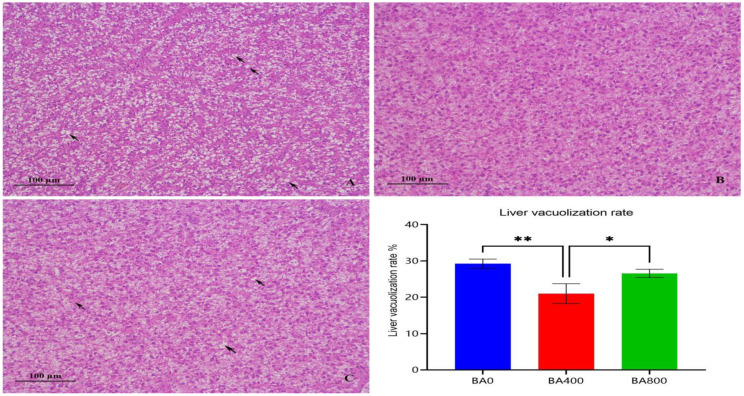
Liver tissue sections and liver vacuolization rate of *Pelteobagrus fulvidraco*. (**A**–**C**) represent the three groups of BA0, BA400, and BA800. * Significant difference between two groups at *p* < 0.05; ** significant difference between two groups at *p* < 0.01. Black arrows indicate hepatic vacuolization.

In Figure 1, the BA0 group exhibited marked hepatic vacuolization (indicated by black arrows), whereas hepatocytes in the BA400 group were densely arranged with normal morphology. The moderate vacuolization was also observed in the BA800 group. Quantitative analysis revealed significantly reduced hepatic vacuolization area in the BA400 group compared to the BA0 group (*p* < 0.05).

**Figure 2:**
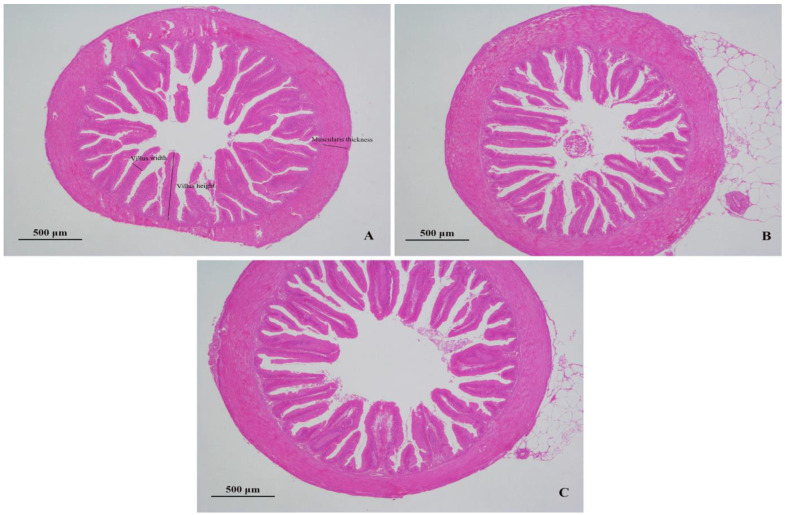
Intestine tissue sections of *Pelteobagrus fulvidraco*; (**A**–**C**) represent the three groups of BA0, BA400, and BA800.

The intestinal villi in the BA400 and BA800 groups were orderly arranged with compact texture compared to the BA0 group (Figure 2). In Table 6, the BA400 group presented significantly greater villus height, villus width, and muscularis thickness than the control (*p* < 0.05), while BA800 displayed no significant differences (*p* > 0.05).

### 3.6. Intestinal Microbiota Analysis

**Figure 3:**
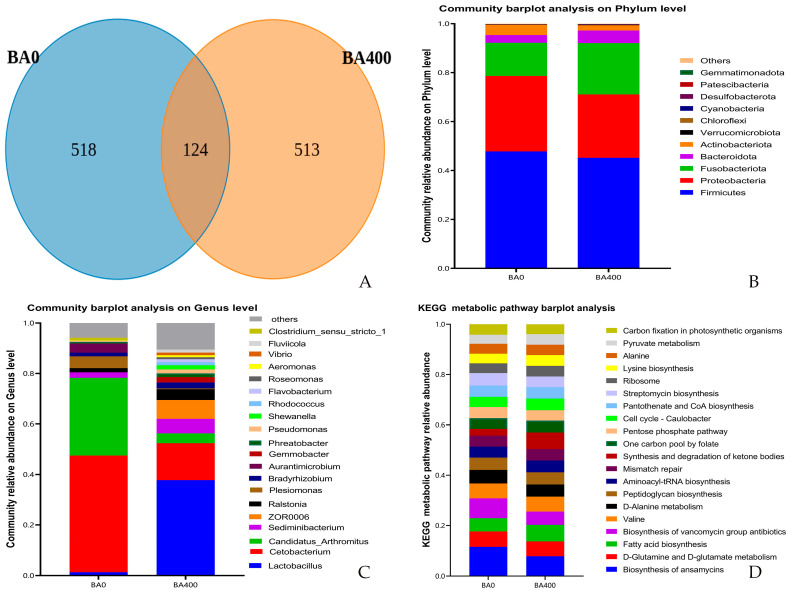
Dietary effects of baicalin on intestinal microbiota of *Pelteobagrus fulvidraco*. (**A**) Venn diagram of OTUs. (**B**) Community barplot analysis on the phylum level. (**C**) Community barplot analysis on the genus level. (**D**) KEGG metabolic pathway barplot analysis.

The coverage values for all groups approached 1, indicating that the microbial community was sufficiently sampled and the obtained data accurately represented the underlying microbial population. As shown in Table 7, the Shannon and Simpson indices of intestinal microbiota in the BA400 group were significantly higher than those in the BA0 group (*p* < 0.05), while no significant differences were observed in Chao1 and ACE (Abundance-based Coverage Estimator) indices between the two groups (*p* > 0.05). A total of 1155 OTUs were identified in the intestinal contents from both groups, with 124 OTUs shared by the two groups, and the BA0 and BA400 groups exhibited 518 and 513 unique OTUs, respectively (Figure 3A).

At the phylum level, the dominant phyla in both BA0 and BA400 groups were *Firmicutes*, *Proteobacteria*, and *Fusobacteriota*. Compared with the BA0 group, the BA400 group showed significantly decreased relative abundances of *Proteobacteria* and *Actinobacteriota* (*p* < 0.05), along with a significant increase in *Fusobacteriota* abundance (*p* < 0.05) (Figure 3B).

At the genus level, the dominant genera in the BA0 group were *Cetobacterium*, *Candidatus_Arthromitus*, and *Plesiomonas*, whereas the BA400 group was dominated by *Lactobacillus*, *Cetobacterium*, and *ZOR0006*. Significant increases in the relative abundances of *Lactobacillus* and ZOR0006 were observed in the BA400 group compared to the BA0 group (*p* < 0.05), while the abundances of *Cetobacterium*, *Candidatus_Arthromitus*, and *Plesiomonas* were significantly reduced (*p* < 0.05) (Figure 3C).

The BA400 group exhibited a significantly enhanced metabolic potential of fatty acid synthesis and ketone body synthesis compared to the BA0 group (*p* < 0.05). Conversely, the synthesis pathways of ansamycins and vancomycin-class antibiotics were significantly downregulated in the BA400 group (*p* < 0.05) (Figure 3D).

## 4. Discussion

### 4.1. Growth Performance

Jia et al. [7] reported that 400 mg/kg dietary baicalin supplementation significantly decreased the FCR in Nile tilapia (*Oreochromis niloticus*). As a structurally analogous flavonoid to baicalin, baicalein (200 mg/kg) was shown to enhance the growth performance of grass carp (*Ctenopharyngodon idella*) [16]. In hybrid grouper (*Epinephelus fuscoguttatus♀× E. lanceolatus♂*), dietary supplementation with 1–3% *Scutellaria baicalensis* extract (containing baicalin) significantly increased the growth, which might be attributed to the upregulation of growth hormone receptor (GHR), insulin-like growth factor I (IGF-I), and IGF-II gene expression, along with the enhanced intestinal digestive enzyme activity and antioxidant capacity [18]. Similarly, 10 g/kg *Scutellaria baicalensis* (the botanical source of baicalin) supplementation decreased the FCR of channel catfish [19]. Consistent with these findings, our trial revealed that 200–400 mg/kg baicalin addition significantly increased WG and reduced FCR in yellow catfish.

Notably, the 800 mg/kg baicalin group showed significantly reduced growth performance compared to the BA400 group, suggesting potential toxicological effects at high doses. This observation aligns with the reports that excessive flavonoid intake may induce pro-oxidant toxicity through free radical-mediated DNA damage or inhibition of DNA-associated enzymes (e.g., topoisomerases) [20], while causing DNA and chromosomal mutations as a mutagen [21]. Notably, the significantly increased feed conversion ratio (FCR) at the 800 mg/kg supplementation level indicates a metabolic impairment in nutrient utilization rather than a taste-mediated avoidance behavior. Similar dose-dependent growth suppression has also been encountered in channel catfish fed with a 15 g/kg *Scutellaria baicalensis* addition [19] and grass carp fed with a 600 mg/kg baicalein addition [17].

### 4.2. Serum, Liver, and Intestinal Biochemical Indices

Alkaline phosphatase (ALP) regulates immune function by promoting the dephosphorylation of bacterial or endogenous pro-inflammatory factors [22]. Acid phosphatase (ACP), primarily located in lysosomes, modulates immunity by dephosphorylating pathogenic substances engulfed by lysosomes [23]. Lysozyme (LZM) exerts bactericidal effects by dissolving peptidoglycan in bacterial cell walls [24]. Catalase (CAT) and superoxide dismutase (SOD) efficiently scavenge free radicals in organisms [23]. Malondialdehyde (MDA), a terminal product of lipid peroxidation, reflects the severity of oxidative stress on biomembrane systems and serves as a critical indicator of oxidative damage [25]. Triglyceride (TG) is a key parameter for assessing blood lipid levels and plays vital roles in lipid metabolism and energy homeostasis in fish.

Baicalin exerts anti-inflammatory and immuno-modulatory effects by regulating the TLRs/MyD88/NF-κB and TLRs/MyD88/MAPK signaling pathways [26]. Bao et al. [2] reported that baicalin combats viral infections through direct virucidal activity, inhibition of viral replication, modulation of host cell functional protein expression, and anti-inflammatory mechanisms, while also showing antibacterial and antifungal properties to enhance disease resistance ability. Previous studies have demonstrated that baicalin protected Nile tilapia infected with *Streptococcus agalactiae* by attenuating bacterial virulence and enhancing non-specific immunity [8] and improved the survival of yellow catfish challenged with *Aeromonas hydrophila* [10]. Furthermore, Li et al. [9] found that baicalin upregulated the expression of crustin, anti-lipopolysaccharide factors (ALFs), and LZM genes in *Macrobrachium rosenbergii* infected with *Vibrio parahaemolyticus*. In this experiment, baicalin significantly increased serum ALP, ACP, and LZM activities, indicating its capacity to enhance non-specific immune responses in yellow catfish.

Baicalin exhibits strong antioxidant activity due to the two phenolic hydroxyl groups, which can scavenge free radicals and reactive oxygen species [27]. Additionally, baicalin can reduce oxidative stress by modulating the Nrf2-Keap1 and p38 MAPK signaling pathways in the liver [2]. Jia et al. [7] demonstrated that baicalin alleviated H_2_O_2_-induced oxidative stress in Nile tilapia by elevating hepatic SOD, total antioxidant capacity (T-AOC), and glutathione (GSH) levels. Yan et al. [10] reported that intraperitoneal injection of baicalin enhanced SOD, GSH, and CAT activities in the liver of yellow catfish infected with *A. hydrophila*. In the present study, baicalin increased T-AOC, CAT, and SOD levels while reducing MDA content in both liver and intestinal tissues, confirming its antioxidant-enhancing properties.

Dietary supplementation with 400 mg/kg baicalin mitigated obesity and hyperlipidemia in mice fed with high-fat diets [28]. Elshopakey et al. [29] observed that baicalin significantly reduced serum triglyceride levels in Nile tilapia, and *Scutellaria baicalensis* extract also decreased serum triglycerides in Far Eastern catfish (*Silurus asotus*) [30]. Consistent with these findings, the present results demonstrated that baicalin effectively lowered serum triglyceride levels in yellow catfish. The reduction is considered beneficial for fish health, as it helps maintain metabolic homeostasis and reduces the risk of lipid-related disorders like hepatic steatosis, as we mentioned below.

### 4.3. Liver and Intestinal Histology

Hepatic vacuolization, typically resulting from excessive lipid accumulation, may impair the normal metabolic functions [31]. Aspartate aminotransferase (AST), a key transaminase in amino acid metabolism, predominantly localizes in the liver and exhibits elevated serum concentrations during hepatic dysfunction [32]. Baicalin could reduce hepatic lipid deposition in mice by activating AMP-activated protein kinase (AMPK) and acetyl-CoA carboxylase (ACC), while downregulating lipogenesis-related genes, including fatty acid synthase and its upstream regulator SREBP-1c [33]. It has been reported that baicalin decreased H_2_O_2_-induced hepatic vacuolization in Nile tilapia [7], and dietary supplementation with *Scutellaria baicalensis* reduced serum AST activity and hepatic vacuolization in channel catfish [19]. In this study, 400 mg/kg baicalin supplementation significantly reduced hepatic vacuolization and serum AST activity in yellow catfish, demonstrating its hepato-protective effects.

Intestinal morphological parameters, including villus width, villus height, and muscularis thickness, directly reflect intestinal health. Villus width and height are closely associated with nutrient absorption capacity [34]. Baicalin has been shown to protect colonic tissue by inhibiting intestinal epithelial cell apoptosis and enhancing tight junction protein expression through suppression of the PI3K/AKT signaling pathway [35]. Zhang et al. [36] reported that dietary supplementation with a *Scutellaria*-containing herbal mixture significantly increased intestinal villus width and height in large yellow croaker (*Larimichthys crocea*). The current experiment revealed that 400 mg/kg baicalin supplementation significantly improved intestinal villus width, villus height, and muscularis thickness in yellow catfish, confirming its efficacy in enhancing intestinal structural integrity.

### 4.4. Intestinal Microbiota

The intestinal microbiota is a vital component of the gut, playing significant roles in nutrient absorption, immune system development, and defense against pathogenic invasions [37]. The biodiversity and community composition of the intestinal microbiota can reflect intestinal health [38]. In this experiment, baicalin significantly enhanced the alpha diversity (Shannon and Simpson indices) of the intestinal microbiota in yellow catfish. At the phylum level, the dominant phyla in both groups were *Firmicutes*, *Proteobacteria*, and *Fusobacteria*, consistent with the previous findings in yellow catfish [39]. Compared to the control group, the baicalin-supplemented group exhibited significantly reduced relative abundances of *Proteobacteria* and *Actinobacteria* and significantly increased the abundance of *Fusobacteria*. *Proteobacteria* contain some pathogenic and opportunistic pathogenic genera that may impair intestinal health in fish [40]. In contrast, *Fusobacteria* can synthesize various vitamins and short-chain fatty acids (SCFAs) [41,42], potentially promoting intestinal health for yellow catfish.

At the genus level, the dominant genera in the control group were *Cetobacterium*, *Candidatus_Arthromitus*, and *Plesiomonas*, whereas the baicalin-supplemented group (400 mg/kg) showed dominance of *Lactobacillus*, *Cetobacterium*, and *ZOR0006*. These results aligned with the previous reports by Chen et al. [39], who identified *Cetobacterium* and *Candidatus_Arthromitus* as dominant genera in yellow catfish, and by Zhu et al. [43], who reported *Cetobacterium* and *Plesiomonas* as key genera. Compared to the control, baicalin supplementation significantly increased the relative abundances of *Lactobacillus* and *ZOR0006*, while reducing the relative abundances of *Cetobacterium*, *Candidatus_Arthromitus*, and *Plesiomonas*. *Lactobacillus*, known as a probiotic in fish intestines, is able to regulate the intestinal environment via lactic acid secretion [44]. *ZOR0006*, an intestinal-specific bacterium, participates in carbohydrate decomposition and lactate synthesis [45]. Yan et al. [10] demonstrated that baicalin elevated Chao1 and ACE indices of intestinal microbiota in yellow catfish infected with *Aeromonashydrophila*. Xia et al. [46] found that *Scutellaria baicalensis* extract reduced the harmful *Deltaproteobacteria* level in the intestine of rabbit fish (*Siganus fuscescens*). Similarly, Du et al. [47] reported that baicalin improved intestinal microbial composition in koi carp. Consistent with these previous findings, the present results indicated that baicalin enhanced intestinal microbial richness and positively modulated microbiota composition in yellow catfish.

Analysis of KEGG metabolic pathways derived from intestinal microbiota composition reflects microbial metabolic activity [48]. The fatty acid synthesis in the gut is primarily driven by anaerobic bacterial fermentation of carbohydrates to produce SCFAs [49], which can inhibit harmful bacteria and regulate host immunity [50]. Baicalin has been shown to enhance SCFA synthesis in mice by increasing the abundances of *Firmicutes*, *Bacteroidetes*, and *Fusobacteria* [51]. Xia et al. [46] also observed the elevated SCFA synthesis in the intestines of rabbit fish fed with a *Scutellaria baicalensis* extract-supplemented diet. In this study, the increased fatty acid metabolism in the baicalin group may correlate with the higher abundances of *Fusobacteria* and *Bacteroidetes*. Additionally, *Actinobacteria* can reduce microbial diversity and inhibit probiotic growth through the production of antibiotics, such as ansamycins and vancomycins [52,53,54]. The decreased synthesis of these antibiotics in the present baicalin group likely corresponded to the reduced *Actinobacteria* abundance.

## 5. Conclusions

Dietary supplementation of baicalin significantly improved weight gain, feed utilization, antioxidant capacity, and liver and intestinal tissue morphology in yellow catfish, while positively modulating intestinal microbiota composition. Under the experimental conditions, the optimal dietary supplementation level of baicalin is 400 mg/kg for yellow catfish. These benefits suggest that baicalin could serve as a functional feed additive to promote fish health, growth, and potentially improve product quality in farmed yellow catfish.

Future research should focus on validating the positive effects in practical farming environments and exploring the molecular mechanisms underlying baicalin’s regulatory roles in gut–liver crosstalk, immunity, and nutrient metabolism.

## Figures and Tables

**Table 1:** Formulation and proximate composition of experimental diets (air dry basis, %).

Ingredients	BA0	BA100	BA200	BA400	BA800
Fish meal	18.00	18.00	18.00	18.00	18.00
Chicken meal	12.00	12.00	12.00	12.00	12.00
Soybean meal	20.00	20.00	20.00	20.00	20.00
Soybean protein concentrate	6.00	6.00	6.00	6.00	6.00
Wheat flour	22.96	22.95	22.94	22.92	22.88
Corn gluten meal	8.00	8.00	8.00	8.00	8.00
Cottonseed gluten meal	4.00	4.00	4.00	4.00	4.00
Fish oil	1.00	1.00	1.00	1.00	1.00
Soybean oil	2.00	2.00	2.00	2.00	2.00
Soy lecithin	1.30	1.30	1.30	1.30	1.30
Baicalin	0.00	0.01	0.02	0.04	0.08
Ca(H_2_PO_4_)	2.50	2.50	2.50	2.50	2.50
Vitamin and mineral premix ^1^	2.00	2.00	2.00	2.00	2.00
Choline chloride	0.20	0.20	0.20	0.20	0.20
Vitamin C phosphate	0.04	0.04	0.04	0.04	0.04
Total	100.00	100.00	100.00	100.00	100.00
Proximate composition ^2^					
Crude protein	46.53	46.60	46.71	46.52	46.49
Crude lipid	8.95	8.98	8.97	8.97	8.97
Crude ash	8.63	8.98	8.52	8.90	8.69
Moisture	8.36	8.40	8.61	8.33	8.58

^1^ Vitamin and mineral content in premix per kilogram: VA 200,000 IU, VD_3_ 12,000 IU, VE 1600 mg, VK_3_ 930 mg, VB_1_ 280 mg, VB_2_ 380 mg, VB_6_ 350 mg, VB_12_ 0.02 mg, and nicotinamide 2500 mg; D-calciumpantothenate 1200 mg, folic acid 2 mg, inositol 100 mg, and biotin 0.2 mg; Fe 540 mg, Zn 830 mg, Mn 500 mg, Cu 200 mg, Co 1 mg, Se 0.35 mg, and I 1 mg. ^2^ Nutrient levels were all measured values.

**Table 2:** Dietary effects of baicalin on growth performance and body parameters of *Pelteobagrus fulvidraco*.

Items	BA0	BA100	BA200	BA400	BA800
Initial weight (g)	11.23 ± 0.05	11.20 ± 0.04	11.20 ± 0.07	11.27 ± 0.08	11.09 ± 0.04
Final weight (g)	31.82 ± 0.25 ^a^	32.57 ± 0.80 ^a^	36.30 ± 1.14 ^bc^	39.10 ± 1.08 ^c^	33.23 ± 1.27 ^ab^
Survival (%)	95.56 ± 3.85 ^a^	100 ± 0.00 ^b^	100 ± 0.00 ^b^	100 ± 0.00 ^b^	100 ± 0.00 ^b^
WG (%)	183.28 ± 3.44 ^a^	190.77 ± 7.16 ^a^	228.79 ± 11.14 ^bc^	247.04 ± 9.62 ^c^	199.43 ± 12.74 ^ab^
SGR (%)	1.75 ± 0.05 ^a^	1.88 ± 0.06 ^a^	2.12 ± 0.06 ^bc^	2.22 ± 0.05 ^c^	1.96 ± 0.08 ^ab^
FCR	1.80 ± 0.02 ^a^	1.71 ± 0.07 ^a^	1.42 ± 0.06 ^b^	1.32 ± 0.06 ^b^	1.66 ± 0.09 ^a^
CF (g/cm^3^)	1.75 ± 0.28	1.84 ± 0.16	1.74 ± 0.15	1.82 ± 0.10	1.81 ± 0.29
VSI (%)	8.83 ± 0.82	7.87 ± 2.56	8.46 ± 1.18	7.93 ± 0.60	8.86 ± 1.44
HSI (%)	2.56 ± 0.33	2.52 ± 0.10	2.31 ± 0.32	2.36 ± 0.16	2.34 ± 0.29

Data with different letters in the same row indicate a significant difference (*p* < 0.05). The same applies to the tables below.

**Table 3:** Dietary effects of baicalin on serum biochemical indices of *Pelteobagrus fulvidraco*.

Items	BA0	BA100	BA200	BA400	BA800
TCHO (mmol/L)	8.11 ± 0.80	7.04 ± 0.04	7.68 ± 0.33	6.99 ± 0.52	7.95 ± 0.55
TG (mmol/L)	10.68 ± 1.11 ^ab^	9.11 ± 0.41 ^c^	9.41 ± 0.79 ^bc^	9.19 ± 0.79 ^c^	10.87 ± 0.84 ^a^
ALP (U/L)	67.32 ± 3.46 ^a^	66.51 ± 6.25 ^a^	73.94 ± 6.64 ^a^	90.43 ± 8.73 ^b^	74.06 ± 5.66 ^a^
ACP (U/L)	286.03 ± 17.10 ^a^	296.00 ± 18.12 ^ab^	299.24 ± 17.66 ^ab^	314.12 ± 15.11 ^b^	304.47 ± 17.51 ^ab^
ALT (U/L)	4.39 ± 0.32	4.67 ± 0.19	4.41 ± 0.16	4.55 ± 0.27	4.46 ± 0.14
AST (U/L)	140.38 ± 11.42 ^a^	101.43 ± 14.04 ^bc^	81.32 ± 1.67 ^c^	87.63 ± 1.67 ^c^	110.00 ± 12.38 ^b^
TP (gprot/L)	31.61 ± 1.10	29.82 ± 2.02	30.58 ± 1.58	30.32 ± 2.52	29.42 ± 1.92
LZM (ug/mL)	1.18 ± 0.19 ^a^	1.39 ± 0.17 ^a^	1.79 ± 0.19 ^b^	2.36 ± 0.23 ^c^	1.51 ± 0.26 ^ab^

Data with different letters in the same row indicate a significant difference (*p* < 0.05).

**Table 4:** Dietary effects of baicalin on antioxidant indices in the liver and intestine of *Pelteobagrus fulvidraco*.

Items	BA0	BA100	BA200	BA400	BA800
Liver					
T-AOC (mmol/mg prot)	0.16 ± 0.05 ^a^	0.23 ± 0.04 ^bc^	0.26 ± 0.04 ^cd^	0.31 ± 0.05 ^d^	0.19 ± 0.04 ^ab^
MDA (nmol/mgprot)	4.44 ± 0.58 ^a^	2.96 ± 0.76 ^b^	2.50 ± 0.36 ^b^	2.35 ± 0.58 ^b^	2.91 ± 0.41 ^b^
CAT (U/mgprot)	8.70 ± 1.91 ^a^	11.93 ± 2.26 ^ab^	13.00 ± 1.90 ^bc^	15.71 ± 2.31 ^c^	11.56 ± 2.26 ^ab^
SOD (U/mgprot)	71.81 ± 10.11 ^a^	94.69 ± 10.01 ^b^	109.58 ± 4.38 ^c^	140.86 ± 5.33 ^d^	109.74 ± 6.08 ^c^
Intestine					
T-AOC (μmol/mg prot)	0.017 ± 0.004 ^a^	0.022 ± 0.003 ^ab^	0.026 ± 0.004 ^b^	0.025 ± 0.004 ^b^	0.019 ± 0.003 ^ab^
MDA (nmol/mgprot)	1.50 ± 0.26 ^a^	0.76 ± 0.20 ^b^	0.72 ± 0.14 ^b^	0.79 ± 0.19 ^b^	1.17 ± 0.28 ^ab^
CAT (U/mgprot)	18.08 ± 1.58 ^a^	25.55 ± 5.08 ^ab^	23.71 ± 4.98 ^ab^	31.21 ± 3.76 ^b^	20.84 ± 3.36 ^a^
LZM (ug/mgprot)	0.06 ± 0.01	0.064 ± 0.01	0.060 ± 0.01	0.079 ± 0.009	0.070 ± 0.01
SOD (U/mgprot)	6.99 ± 2.38 ^a^	8.12 ± 2.39 ^a^	14.43 ± 2.30 ^b^	16.56 ± 1.25 ^b^	9.84 ± 2.93 ^a^

Data with different letters in the same row indicate a significant difference (*p* < 0.05).

**Table 5:** Dietary effects of baicalin on the body proximate composition of *Pelteobagrus fulvidraco* (wet weight, %).

Items	BA0	BA100	BA200	BA400	BA800
Moisture	72.38 ± 0.40	72.12 ± 1.08	72.25 ± 0.70	72.00 ± 1.09	71.65 ± 0.78
Crude ash	4.06 ± 0.53	4.00 ± 0.23	4.08 ± 0.21	3.95 ± 0.05	4.07 ± 0.20
Crude lipid	6.48 ± 0.46	6.61 ± 0.46	6.51 ± 0.36	6.46 ± 0.59	6.37 ± 0.45
Crude protein	15.33 ± 0.64	15.64 ± 0.68	15.33 ± 0.73	15.60 ± 0.67	15.89 ± 0.26

**Table 6:** Dietary effects of baicalin on intestinal tissue morphology of *Pelteobagrus fulvidraco*.

Items (μm)	BA0	BA400	BA800
Villus height	570.4 ± 20.13 ^a^	670.9 ± 51.51 ^b^	561.2 ± 31.65 ^a^
Villus width	105.6 ± 5.44 ^a^	138.4 ± 19.65 ^b^	120.8 ± 14.78 ^ab^
Muscularis thickness	167.5 ± 26.05 ^a^	222.6 ± 15.29 ^b^	197.1 ± 23.30 ^ab^

Data with different letters in the same row indicate a significant difference (*p* < 0.05).

**Table 7:** Dietary effects of baicalin on the alpha diversity index of intestinal microbiota of Pelteobagrus fulvidraco.

Items	BA0	BA400
Shannon	3.01 ± 0.12 ^a^	4.69 ± 0.007 ^b^
Simpson	0.64 ± 0.03 ^a^	0.89 ± 0.006 ^b^
Chao1	265.53 ± 12.58	299.22 ± 26.85
ACE	266.25 ± 12.24	299.70 ± 26.89
Coverage	0.999 ± 0.00	0.999 ± 0.00

Data with different letters in the same row indicate a significant difference (*p* < 0.05).

## Data Availability

Data are contained within the article.
